# Supplementary material for: Vitamin D Modified DSS-Induced Colitis in Mice via STING Signaling Pathway
Source: Biology (Basel). 2025 Jun 18;14(6):715. doi: 10.3390/biology14060715 (PMC12190092; doi:10.3390/biology14060715)
Supplement: Supplementary file 1 [file biology-14-00715-s001.zip › Supplementary Materials S1: 16s-report of gut microbiota/Groups/diff_analysis/metagenomeRKEGG/figure/boxplot/top10.pdf]

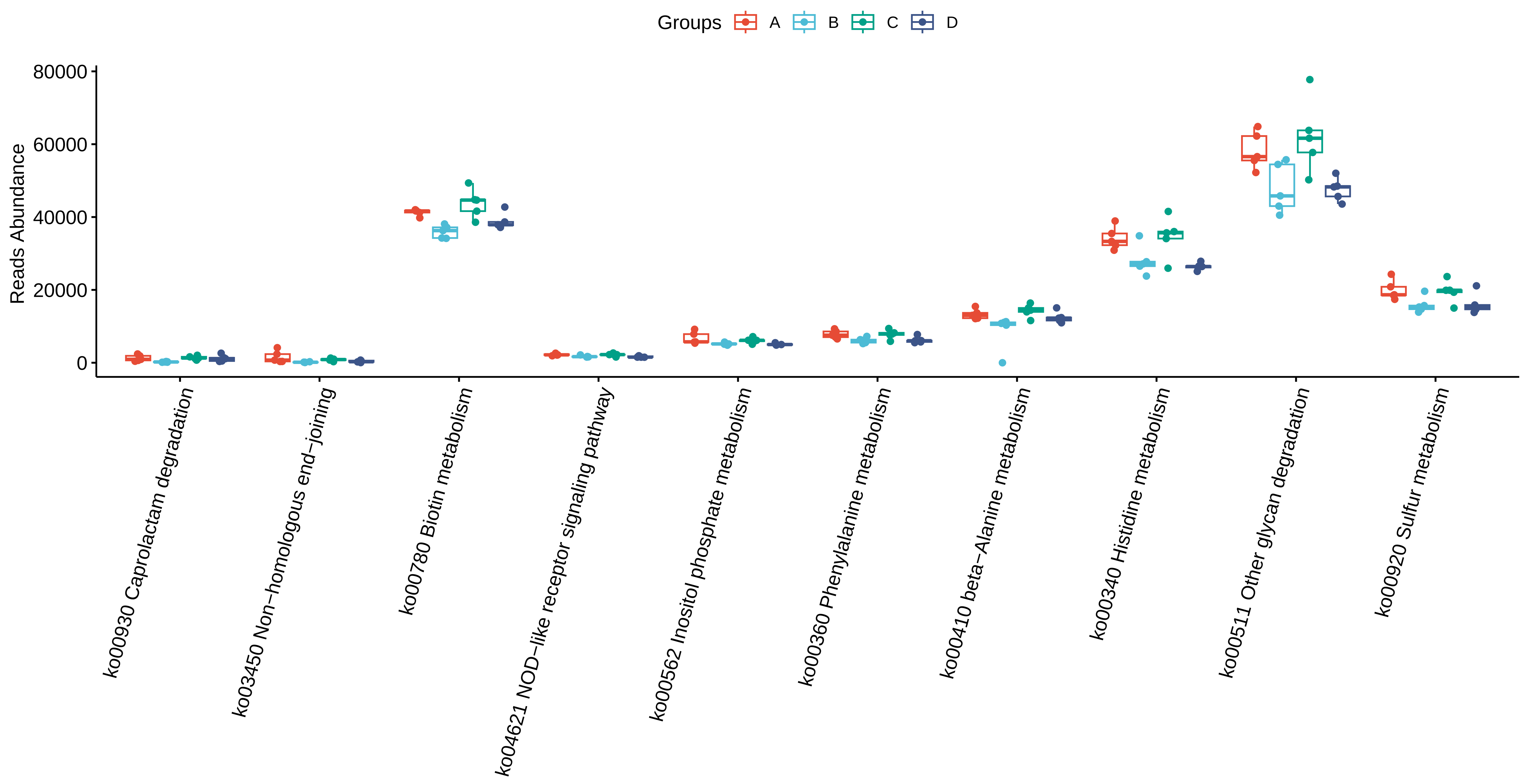

Groups
A
B
C
D
Reads Abundance
80000
60000
40000
20000
0
ko00930 Caprolactam degradation
ko03450 Non−homologous end−joining
ko00780 Biotin metabolism
ko04621 NOD−like receptor signaling pathway
ko00562 Inositol phosphate metabolism
ko00360 Phenylalanine metabolism
ko00410 beta−Alanine metabolism
ko00340 Histidine metabolism
ko00511 Other glycan degradation
ko00920 Sulfur metabolism
